# Beyond the Surface: A Unique Presentation of Acute Promyelocytic Leukemia as Idiopathic Intracranial Hypertension: A Case Report

**DOI:** 10.1002/cnr2.70064

**Published:** 2024-11-26

**Authors:** Amirhossein Mirhosseini, Mirmassoud Shariat, Mandana Ebrahimi, Negar Saeedy, Behnaz Nouri

**Affiliations:** ^1^ Department of Clinical Hematology and Oncology, Faculty of Medicine Alborz University of Medical Sciences Karaj Iran; ^2^ Neurology Department, Clinical Research Development Unit of Imam Ali Hospital Alborz University of Medical Sciences Karaj Iran; ^3^ Department of Internal Medicine, School of Medicine Alborz University of Medical Sciences Karaj Iran; ^4^ Student Research Committee, Clinical Research Development Unit of Imam Ali Hospital Alborz University of Medical Sciences Karaj Iran

**Keywords:** acute promyelocytic leukemia, all‐trans‐retinoic acid, idiopathic intracranial hypertension, pseudotumor cerebri (ptc)

## Abstract

**Background:**

Acute promyelocytic leukemia (APL) is a subtype of acute myeloid leukemia defined by a fusion gene transcript called PML::RARA. Pseudotumor cerebri (PTC) is characterized by elevated intracranial pressure (ICP) and presents with pulsatile tinnitus, headaches, and visual disturbances. Pseudotumor cerebri has been observed in some patients undergoing long‐term ATRA therapy. While both APL and PTC have been individually described in the literature, their coexistence in a single patient, especially with PTC being the first clinical manifestation of APL, is an exceptionally rare occurrence, making this case particularly unique and warranting further investigation.

**Case Presentation:**

We present the case of a 30‐year‐old woman who initially presented with symptoms suggestive of PTC. The diagnosis of PTC was confirmed through a lumbar puncture. However, further investigations revealed that the patient had APL, which is typically sensitive to treatment with all‐trans‐retinoic acid (ATRA). To our knowledge, the association between APL and PTC as the first clinical manifestation of APL in adult patients has not been previously reported in the literature, further highlighting the exceptional rarity of this case.

**Conclusions:**

This case underscores the importance of considering underlying systemic diseases in patients presenting with PTC, as it may represent an exceptionally rare initial manifestation of a rare underlying malignancy such as APL. The coexistence of APL and PTC in this patient highlights the need for a comprehensive evaluation and management approach to promptly diagnose and treat coexisting conditions. Healthcare providers must be aware of this association and consider the possibility of APL when evaluating patients with PTC symptoms. Such awareness can prompt timely recognition and appropriate management, thereby improving patient outcomes and preventing potential delays in diagnosis and treatment.

## 1 Introduction

Acute promyelocytic leukemia (APL) is a rare subtype of acute myeloid leukemia, characterized by significant bleeding tendencies and the presence of promyelocytes in the bone marrow and blood. The genetic hallmark of APL is the balanced reciprocal translocation t(15;17)(q22;q12), which fuses promyelocytic leukemia (PML) and retinoic acid receptor alpha (RARA) genes, leading to leukemia by disrupting normal myeloid development. Patients with APL typically present with symptoms such as weakness, bleeding, visual abnormalities, and thrombotic complications. The coagulopathy associated with APL is life‐threatening, necessitating emergency care [1]. In the mid‐1980s, ATRA monotherapy was introduced, showing high response rates but short response durations [2]. The subsequent introduction of combination therapies involving ATRA, chemotherapy, and arsenic trioxide has drastically improved the prognosis of this once‐fatal condition. Currently, advancement is occurring at a dizzying rate across all leukemias.

In idiopathic intracranial hypertension (IIH), idiopathic implies the exclusion of other possible causes for intracranial hypertension, which have been uncovered during history, physical examination, and investigations such as neuroimaging and lumbar puncture exams. Obese women of childbearing age are particularly susceptible to it, typically presenting with symptoms such as pulsatile tinnitus, visual impairment, or headaches [3]. Treatment options include weight loss, medication, and, in some cases, surgery.

The case of this 30‐year‐old woman, initially diagnosed with IIH but was later found to have APL, underscores the critical importance of thorough investigation in patient care. Investigating underlying systemic disorders in patients presenting with IIH is vital, as it can prevent delays in recognizing and treating comorbid conditions, potentially saving lives.

### 1.1 Case Presentation

In February 2023, a 30‐year‐old Iranian woman was admitted to the emergency room at Imam Ali Hospital, presenting with a four‐day history of blurred vision, general fatigue, and occasional headaches. She had also developed ecchymoses on her left arm, both thighs, and the posterior compartments of her legs. The patient reported no neck stiffness, photophobia, fever, or altered mental state. She had a previous delivery 6 months prior to her current presentation, with complications such as gestational hypertension and gestational diabetes mellitus.

**FIGURE 1:**
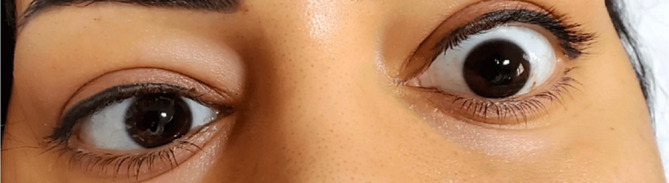
The figure reveals the patient's marked inability to abduct her eyes, which indicates sixth nerve palsy. This finding highlights the neurological involvement associated with the patient's condition.

Upon admission, the patient was hemodynamically stable. Her right eye's visual acuity was limited to hand motion, and she exhibited a bilateral inability to abduct her eyes, indicating sixth or abducens nerve palsy (Figure 1).

**FIGURE 2:**
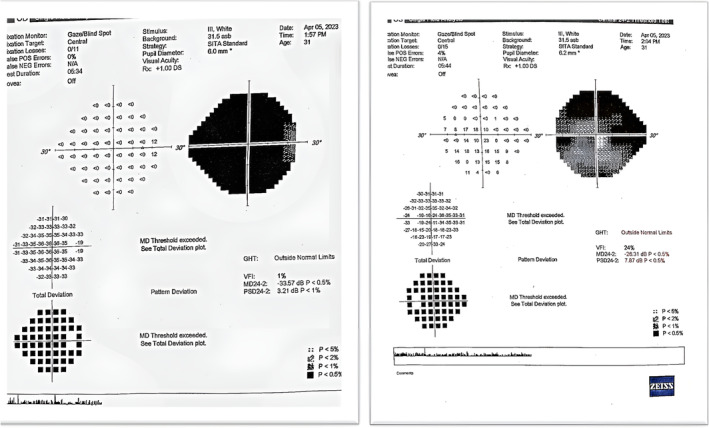
Comprehensive perimetry examination conducted during initial hospitalization. The right figure is the result for the left eye and the left figure is the result for the left eye. It revealed the pattern of vision loss in the left and right eyes, predominantly in the right eye.

Examination of the visual fields showed bilateral papilledema. Figure 2 depicts the results of the perimetry examination conducted for the patient.

Her laboratory data indicated pancytopenia, with a white blood cell count of 0.95 × 10^9^/L, a hemoglobin level of 51 g/L, and platelets of 24 × 10^9^/L. The coagulation test revealed a fibrinogen level of 3.46 g/L, prothrombin time of 18.8 s, and activation partial thromboplastin time ratio of 22. The D‐dimer was 41.9 mg/L.

Table 1 comprehensively reviews the patient's laboratory data upon admission, comprising all pertinent test results.

**TABLE 1:** Comprehensive laboratory data upon admission.

	Test	Result	Reference value	Unit
Routine hematology	WBC	0.95	4.23–10.2	×10^9^/L
	Segment	30.6	34–71	%
	Lymphocyte	54.7	19–51	%
	Monocyte	14.7	4–12	%
	N. RBC	9.5	0	%
	RBC	1.55	4.2–5.4	10^12^/L
	Hb	51	120–160	g/L
	Hct	13.9	36–46	%
	MCV	89.7	80–100	fL
	Platelet	24	150–450	×10^9^/L
	RET	1.4	0.8–2.5	%
RBC Morphology	Anisocytosis	1+	0	—
	Microcytes	1+	0	—
	Hypochromic cell	1+	0	—
	Ovalocyte	2+	0	—
	Spherocyte	Mild	0	—
Coagulation test	PT	18.8	11–13.5	Sec
	aPTT	22	24–36	Sec
	INR	1.6	0.9–1.1	Index
Iron profile	TIBC	355.6	250–450	Mcg/dL
	Serum Iron	285.2	30–150	Mcg/dL
	Ferritin	950	20–110	Mcg/L
CSF analysis	Color	Colorless	—	—
	Appearance	Clear	—	—
	WBC	3	0	
	RBC	1000	0	
	Glucose	60	50–75	
	Total protein	20.5	15–45	Mg/dL
	Neutrophil	0%	0	%
	Lymphocyte	100%	0	%
Autoantibodies	ANA	0.04	< 0.9	S/Co
	Anti‐ds‐DNA	7.94	< 16	IU/mL

Abbreviations: ANA: antinuclear antibody, Anti‐ds‐DNA Ab: anti‐double stranded deoxyribonucleic acid antibodies; aPTT: activated partial thromboplastin time; CSF: cerebrospinal fluid; Hb: hemoglobin; Hct: hematocrit; INR: international normalized ratio; MCV: mean corpuscular volume; N. RBC: nucleated red blood cell; PT: prothrombin time, RBC: red blood cell; WBC: white blood cell.

Following a lumbar puncture, an opening pressure of 50 cmH_2_O was detected, consistent with IIH.

Brain MRI indicated symptoms of elevated intracranial pressure but no other abnormalities.

**FIGURE 3:**
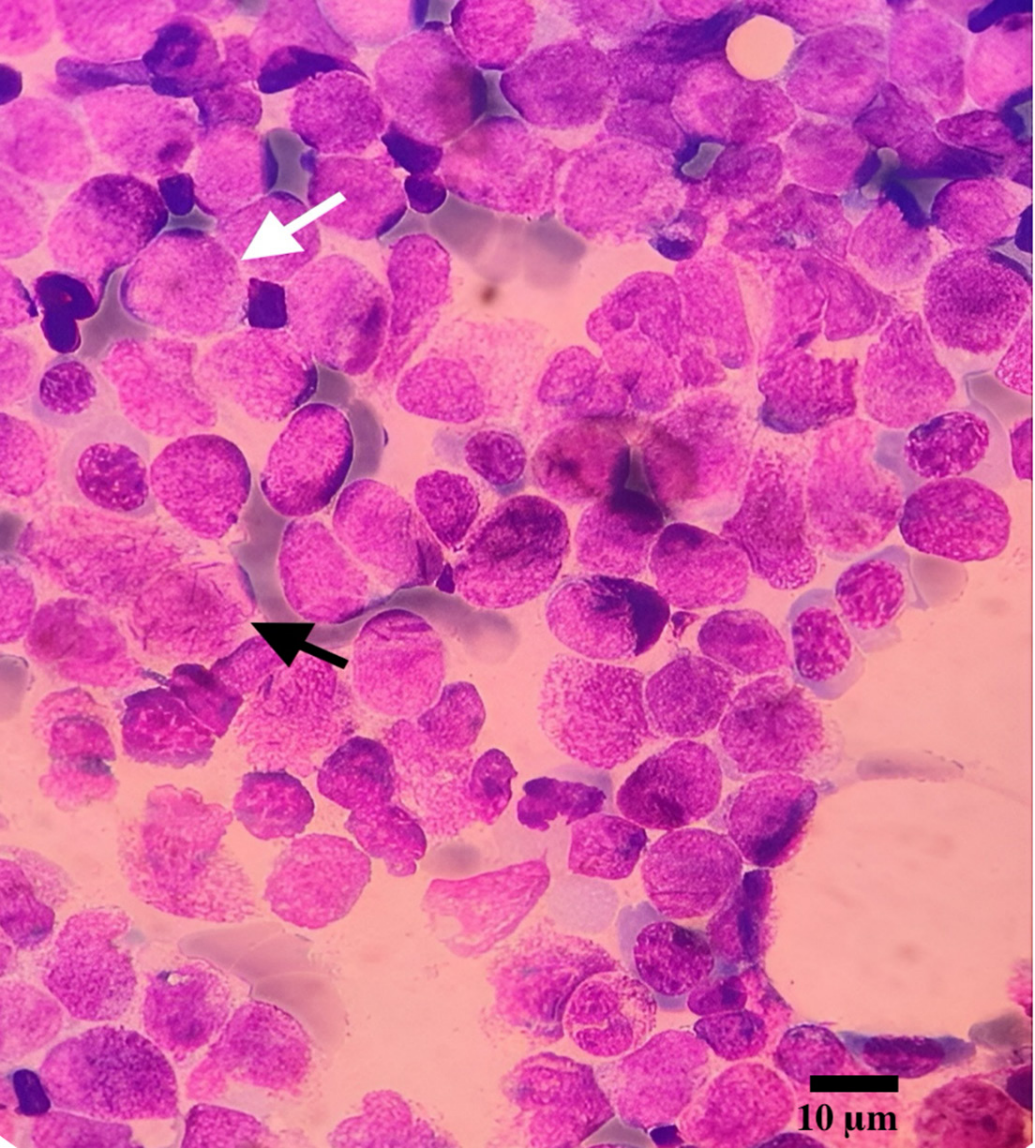
Microphotograph of bone marrow aspirate showing some abnormal promyelocytes and faggot cells, characteristic of acute promyelocytic leukemia (APL), with a focus on one abnormal promyelocyte (white arrow) and one faggot cell (black arrow). Faggot cells are leukemic promyelocytes with multiple clusters of Auer rods in the cytoplasm. The presence of these cells is indicative of an APL diagnosis (Wright‐Giemsa stain, scale bar = 10 μm, magnification ×100).

The morphological evaluation of peripheral blood smear indicated blasts with aberrant promyelocytes, consistent with APL. However, due to the pancytopenia, a bone marrow biopsy was indicated to investigate any potential underlying hematologic disorders. Given the patient's age and absence of other common symptoms, the APL diagnosis was unexpected but confirmed by a bone marrow biopsy (Figure 3). The diagnosis of IIH was the first manifestation of APL.

Two cerebrospinal fluid (CSF) samples were submitted for flow cytometry to rule out involvement in the central nervous system, both of which were negative.

The administration of ATRA at a dose of 45 mg/m^2^ was initially started as part of the treatment for APL. Following a week of treatment with ATRA and arsenic trioxide, the patient's IIH diagnosis was confirmed. ATRA is known to be associated with the development of IIH due to its potential to induce fluid retention and vascular leak syndrome; therefore, it was temporarily discontinued for this reason. However, after a week, the CSF pressure remained high at 47 cm H_2_O. Despite this temporary discontinuation, the patient's CSF pressure remained elevated, necessitating a reassessment of the treatment approach. The oncology team decided to resume ATRA treatment based on the absence of alternative causes for the elevated ICP and the crucial role of ATRA in treating APL. This decision was made after carefully considering the patient's condition and the potential risks and benefits of the treatment options.

Following treatment, the patient received a bone marrow aspiration and biopsy 28 days later, demonstrating a complete morphological response with blasts < 5%.

However, following the administration of consolidation chemotherapy, the presence of PML::RARA became undetectable. Consolidation therapy plays a vital role in post‐remission treatment for acute myeloid leukemia. It is universally acknowledged as a crucial component in therapeutic strategies to achieve long‐term survival.

The patient received supportive care throughout her hospitalization, including blood transfusions and treatment for her headaches, and was briefed on the potential side effects of ATRA. She was discharged with a prescription for ATRA and instructions to follow up with the neurology and hematology departments.

**FIGURE 4:**
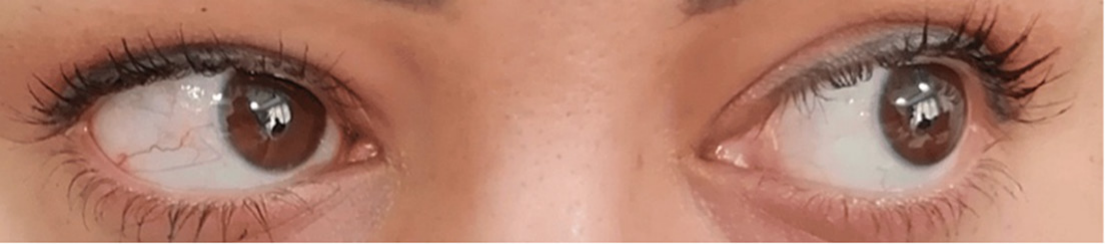
Complete resolution of sixth nerve palsy and Vision preservation following successful ventriculoperitoneal shunting.

Despite the ongoing administration of acetazolamide, the patient's abducens nerve paralysis, headache, and excessive opening pressure persisted. After meeting with a neurosurgeon, it was concluded that a ventriculoperitoneal shunting procedure was necessary. Following the treatment, the patient's headache and abducens nerve palsy fully resolved, ultimately saving her vision (Figure 4).

The patient's overall prognosis was favorable, with a fair possibility of achieving complete remission with appropriate treatment. This case underscores the importance of thorough evaluation in patients with atypical symptoms and unexpected diagnoses and the need to monitor and manage potential treatment‐related adverse effects.

## 2 Discussion

Elevated ICP characterizes idiopathic intracranial hypertension (IIH) without any discernible cause. If not handled promptly, it can lead to lifelong visual loss and neurological problems. IIH is diagnosed according to the Modified Dandy Criteria, including a CSF pressure greater than 25 cmH_2_O and excluding anatomical or vascular abnormalities that might cause elevated ICP [4].

APL denotes the growth of myeloid cells in the bone marrow, which results in bone marrow insufficiency. Generalized weakness, exhaustion, gingival bleeding, petechiae/ecchymoses, vision anomalies resulting from retinal hemorrhages, epistaxis, menorrhagia, and infections are some common manifestations of APL.

The presented case highlights several significant findings and challenges, most notably the diagnosis of APL manifesting initially as IIH. To our knowledge, this is the first reported adult case of APL initially presenting with IIH. This underscores the necessity of thorough diagnostic evaluation in patients presenting with unusual symptom combinations.

First, the most significant challenge in this case was identifying the underlying cause of the patient's IIH. The lack of typical symptoms associated with APL, such as significant bleeding or infection, combined with the postpartum context, initially obscured the diagnosis. The persistence of elevated ICP despite treatment for IIH further complicated the clinical picture, necessitating the discontinuation and subsequent resumption of ATRA therapy, which itself can exacerbate IIH.

Comparing this study with previous cases reveals that while ocular signs are a recognized manifestation of leukemia, the presentation of APL solely with IIH in adults is unprecedented.

The actual mechanism behind APL‐induced IIH is yet to be appropriately established. Literature reviews have proposed several hypotheses on similar topics to elucidate this mechanism.

One of them involves retinoic acid syndrome, where the commonly used ATRA to treat APL can trigger a syndrome characterized by fluid retention and vascular leak syndrome, with IIH being documented as a complication of ATRA syndrome [5].

Another hypothesis revolves around coagulopathy. APL is associated with a coagulopathy that can progress into disseminated intravascular coagulation (DIC), leading to the formation of microthrombi in the cerebral vasculature. This process can result in cerebral ischemia, subsequent edema, elevated ICP, and the onset of IIH [1, 6, 7, 8, 9].

Additionally, leukemic infiltration has been proposed as a contributing factor to APL‐induced IIH. APL can penetrate the central nervous system, affecting tissues such as the meninges or cerebral parenchyma. This infiltration may lead to the restriction of CSF flow, ultimately increasing ICP. The marked elevation in white blood cell count usually observed in APL is considered the fundamental cause for this rise in ICP. Notably, researchers have established a linear association between resistance to CSF outflow and the associated ICP values [10, 11].

Furthermore, direct optic nerve infiltration by APL cells has been suggested as another mechanism linked to the development of IIH. APL cells infiltrating the optic nerve can lead to optic nerve edema, consequently contributing to the development of IIH [12].

Lastly, an additional hypothesis involves elevated ICP due to leukostasis. Leukemic cells congregating in the cerebral vasculature can lead to leukostasis and an increase in ICP, potentially inducing IIH [13].

These hypotheses provide valuable insights into the potential mechanisms underlying APL‐induced IIH, illuminating this condition's complex pathophysiology. However, further research is needed to fully understand these mechanisms and develop targeted therapeutic strategies for such rare presentations.

It is worth mentioning that although postpartum headache is a potential symptom of IIH, it is frequently described during the first 6 weeks following birth rather than 6 months [14]. Therefore, it is exceedingly improbable that postpartum variables alone are the cause of IIH in patients who appear with symptoms after several months.

## 3 Conclusions

This case underscores the importance of early recognition and treatment of APL in patients presenting with atypical symptoms of IIH, particularly when accompanied by hematologic abnormalities like pancytopenia. Healthcare practitioners should maintain a high index of suspicion and pursue comprehensive laboratory investigations when common etiologies for IIH are ruled out. The case also highlights the need for careful monitoring of treatment side effects, such as those associated with ATRA, and the potential necessity for interventions like ventriculoperitoneal shunting in managing persistent IIH. Further research is warranted to elucidate the precise mechanisms of APL‐induced IIH and to develop targeted therapeutic strategies for such rare presentations.

## Author Contributions

**Amirhossein Mirhosseini:** conceptualization, methodology, software, data curation, investigation, validation, formal analysis, supervision, funding acquisition, visualization, project administration, resources, writing – original draft, writing – review and editing. **Mirmassoud Shariat:** conceptualization, methodology, software, data curation, investigation, validation, visualization, writing – review and editing. **Mandana Ebrahimi:** methodology, conceptualization, resources, project administration, investigation. **Negar Saeedy:** conceptualization, methodology, resources, project administration, investigation. **Behnaz Nouri:** conceptualization, methodology, software, data curation, investigation, validation, formal analysis, visualization, project administration, resources, writing – original draft, writing – review and editing.

## Ethics Statement

Since this is a case report, there is no need for ethical approval. Written informed consent was obtained from the patient to publish this case report and accompanying images.

## Consent

Written informed consent was obtained from the patient to publish this case report and accompanying images.

## Conflicts of Interest

The authors declare no conflicts of interest.

## Data Availability

Data sharing is not applicable to this article as no new data were created or analyzed in this study.
